# Revolutionizing biomedical research: The imperative need for heart–kidney-connected organoids

**DOI:** 10.1063/5.0190840

**Published:** 2024-02-27

**Authors:** Sun-Sook Song, Hun-Jun Park, Yong Kyun Kim, Sun-Woong Kang

**Affiliations:** 1 Research Group for Biomimetic Advanced Technology, Korea Institute of Toxicology, Daejeon 34114, Republic of Korea; 2 College of Pharmacy, Chungnam National University Daejeon 34134, Republic of Korea; 3 Department of Biomedicine and Health Sciences, The Catholic University of Korea, Seoul 06591, Republic of Korea; 4 Division of Cardiology, Department of Internal Medicine, Seoul St. Mary's Hospital, The Catholic University of Korea, Seoul 06591, Republic of Korea; 5 Cell Death Disease Research Center, College of Medicine, The Catholic University of Korea, Seoul 06591, Korea; 6 Department of Internal Medicine, College of Medicine, The Catholic University of Korea, St. Vincent's Hospital, Suwon, Republic of Korea; 7 Human and Environmental Toxicology Program, University of Science and Technology, Daejeon 34114, Republic of Korea

## Abstract

Organoids significantly advanced our comprehension of organ development, function, and disease modeling. This Perspective underscores the potential of heart–kidney-connected organoids in understanding the intricate relationship between these vital organs, notably the cardiorenal syndrome, where dysfunction in one organ can negatively impact the other. Conventional models fall short in replicating this complexity, necessitating an integrated approach. By co-culturing heart and kidney organoids, combined with microfluidic and 3D bioprinting technologies, a more accurate representation of *in vivo* conditions can be achieved. Such interconnected systems could revolutionize our grasp of multi-organ diseases, drive drug discovery by evaluating therapeutic agents on both organs simultaneously, and reduce the need for animal models. In essence, heart–kidney-connected organoids present a promising avenue to delve deeper into the pathophysiology underlying cardiorenal disorders, bridging existing knowledge gaps, and advancing biomedical research.

## I. INTRODUCTION

In recent years, the transformative advent of organoid research has rewritten the paradigms of biomedical science. These miniature, simplified versions of organs, known as organoids, are derived from stem cells or tissue-specific progenitors.^1–3^ They are not merely clusters of cells; organoids emulate the complex three-dimensional architecture and multifaceted functionality of actual human organs.^4^ Their development marks a leap forward from two-dimensional cell cultures, which lack the intricate interactions and organization of cells in living tissues.^5^

The necessity for organoid research stems from an imperative to overcome the limitations of traditional *in vitro* models and animal studies, which often fail to fully replicate human organ physiology and pathology.^6–8^ While animal models have contributed significantly to our understanding of biological processes and disease mechanisms, there are considerable interspecies differences that limit the translation of findings to human conditions.^9^ Organoids bridge this gap by offering models that are genetically and biologically closer to human organs.^10,11^ Furthermore, the complexity of human organ development and disease is such that it requires a model capable of reflecting the intricate interplay of cells within a structured, three-dimensional space.^12^ Organoids satisfy this need, allowing for the study of organogenesis and offering a platform to investigate complex diseases in ways that were previously not possible.^13,14^ For instance, cancer organoids can mimic the tumor microenvironment, providing new insight into cancer biology and facilitating the development of targeted therapies.^15,16^

The urgency in advancing organoid research is also driven by the quest for personalized medicine. Organoids can be cultivated from patient-derived cells, including induced pluripotent stem cells (iPSCs), providing a personalized model to study individual responses to drugs and to screen for the most effective treatments.^17–20^ This personalized approach is particularly promising for rare diseases, where patient-specific models may be the only way to explore disease mechanisms and test new treatments. Moreover, the growing prevalence of complex, chronic diseases that affect multiple organ systems, such as diabetes and cardiovascular diseases, necessitates the use of organoids to understand how different tissues interact under healthy conditions and in states of disease.^21,22^ The need is pressing for innovative models like organoids that can mirror these conditions faithfully, thereby illuminating paths to new treatments and potentially cures.

## II. THE CRITICAL INTERPLAY: HEART AND KIDNEYS

The heart and kidneys are not only vital organs in isolation but are also intertwined in their physiological functions, ensuring the body's well-being. Table I gives an overview of complex interplay between the heart and kidneys. The heart, often termed the body's powerhouse, plays the indispensable role of pumping blood throughout the circulatory system.^23^ Every heartbeat ensures that oxygen-rich blood reaches every nook and cranny of the body, from the brain's intricate networks to the toes' extremities.^24^ It is also responsible for receiving deoxygenated blood and sending it to the lungs for reoxygenation.^25^

**TABLE I.** The complex interplay between the heart and kidneys, highlighting their primary functions, contributions to homeostasis, and the potential effects on the other organ in the case of dysfunction.

Organ	Primary function	Contribution to homeostasis	Effect on other in dysfunction
Heart	Central pump of the circulatory system^25–30^	Regulates blood pressure	Reduced blood flow to the kidneys can lead to decreased kidney function
		Distributes oxygen and nutrients	Potentially triggers kidney disorders due to reduced filtration
Kidneys	Filtration and excretion system for waste products^31–35^	Regulates fluid balance	High blood pressure can strain the heart and lead to heart diseases
	Maintains electrolyte balance^36,37^	Filters waste from the bloodstream	Fluid overload may exacerbate heart failure

Conversely, the kidneys, positioned as the body's natural filtration system, continually cleanse the blood of waste products and excess substances.^31,32^ Working tirelessly, they process approximately 180 l of blood daily to produce about 1–2 l of urine.^38,39^ This process helps regulate the body's fluid balance, ensuring that tissues receive the right amount of hydration. The kidneys also play a pivotal role in maintaining electrolyte balance, such as sodium, potassium, and calcium levels, which are critical for cellular functions, nerve conduction, and muscle contraction.^36^

**FIG. 1.**
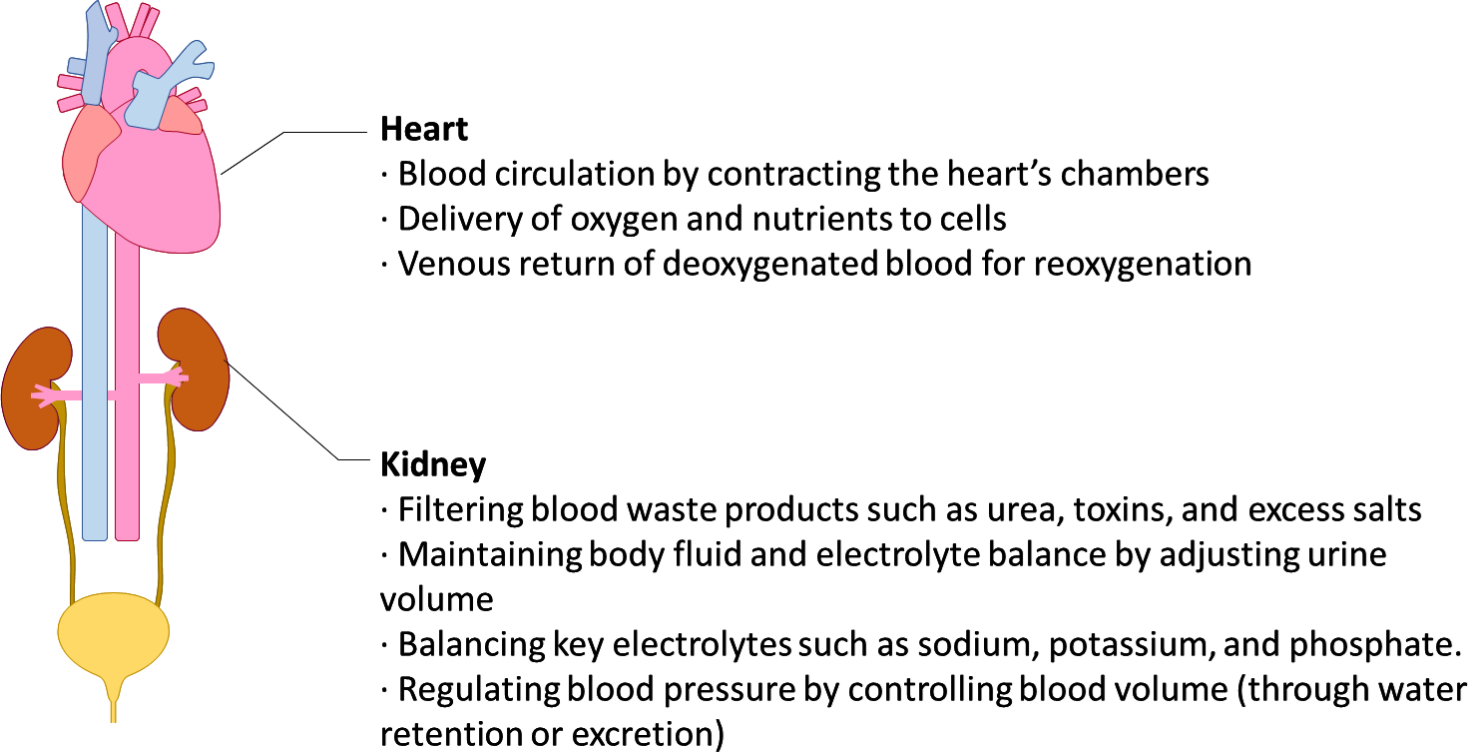
Schematic representation of the role of heart–kidney interplay.

Beyond these primary functions, there exists a complex, bidirectional relationship between the heart and kidneys (Fig. 1). They collaborate closely to regulate blood pressure; the heart adjusts its pumping force based on the blood volume, while the kidneys control volume by altering urine output.^40^ Any alarm in one organ's function can trigger a cascade of events affecting the other. For instance, a weakened heart can reduce blood flow to the kidneys, leading to decreased kidney function, while kidney disorders can lead to fluid buildup, increasing the heart's workload.^41,42^

Emerging research has coined the term “cardiorenal syndrome” to describe this intertwined relationship, wherein a malfunction or disease in one organ can adversely impact the function and health of the other.^43,44^ This connection highlights the importance of studying these organs in tandem rather than isolation, recognizing the profound influence they exert on each other's functionality.

## III. HEART–KIDNEY-CONNECTED ORGANOIDS: A NEW FRONTIER

Organoid research has demonstrated a revolutionary approach to understanding the physiology and pathology of human organs. Organoid technology, particularly with the development of single-organ models, such as heart and kidney organoids, has become a powerful force in biomedical research. These stand-alone systems have enabled groundbreaking studies into individual organ development, disease mechanisms, and therapeutic responses. For example, heart organoids have played a pivotal role in modeling congenital heart diseases and cardiomyopathies, allowing for the recapitulation of cardiac tissue structure and functions *in vitro*.^45^ Researchers have successfully created beating heart organoids that exhibit key aspects of cardiac muscle physiology, providing a dynamic environment to test the cardiotoxic effects of drugs and to study the mechanisms of repair following myocardial injury.^46,47^ Similarly, kidney organoids derived from human pluripotent stem cells have provided profound insight into renal development and disease. These organoids include multiple cell types found in the nephron, the functional unit of the kidney, and are capable of mimicking some of the complex functions of the kidney, including filtration and reabsorption.^48^ They have been central to understanding the pathways involved in polycystic kidney disease and evaluating the nephrotoxic effects of various drug candidates.^49^

However, the exploration of inter-organ relationships, particularly between the heart and kidneys, remains a relatively uncharted domain. In this Perspective, we not only underscore the urgency but also delve into the potential benefits and methodologies behind developing heart–kidney-connected organoids. Even the most advanced 2D cell culture systems and many existing organoid models overlook the complex communication between organs. Such omissions could lead to gaps in our understanding, especially when dealing with multifaceted disorders, such as cardiorenal syndrome. Here lies the potential of heart–kidney-connected organoids. By generating systems that are physically linked and arranged to reflect the physiological proximity *in vivo*, researchers can more accurately model and investigate the subtle interactions between these two vital organs. These heart–kidney-connected organoids are more than mere cell aggregates; they are dynamic and miniaturized representations of organs whose cellular differentiation, spatial orientation, and functional characteristics closely align with their *in vivo* counterparts.^50^ By establishing these connected models, scientists can explore mechanisms that were previously inaccessible or poorly understood. For example, how does a pathological state of the heart impact molecular signaling to the kidneys? Or how do the kidneys respond at a cellular level to cardiac stress? Moreover, these combined organoids provide a unique platform for closely examining shared cellular pathways and uncovering regulatory circuits and molecular cascades that play roles in the health and disease of both organs.

Recognizing that many diseases do not just affect isolated organs but have systemic ramifications, this becomes increasingly important. While advocating for the development of heart–kidney-connected organoids, it is crucial to acknowledge the future challenges. Perfecting co-culture techniques, ensuring that organoids mature and function in tandem, and creating an environment that truly mimics physiological conditions are just some of the hurdles. However, the potential insight to be gained and the leaps toward personalized medicine make this frontier an exciting and necessary venture in modern biomedical research.

## IV. ADVANCED TECHNIQUES FOR IMPROVED INTERACTION

**FIG. 2.**
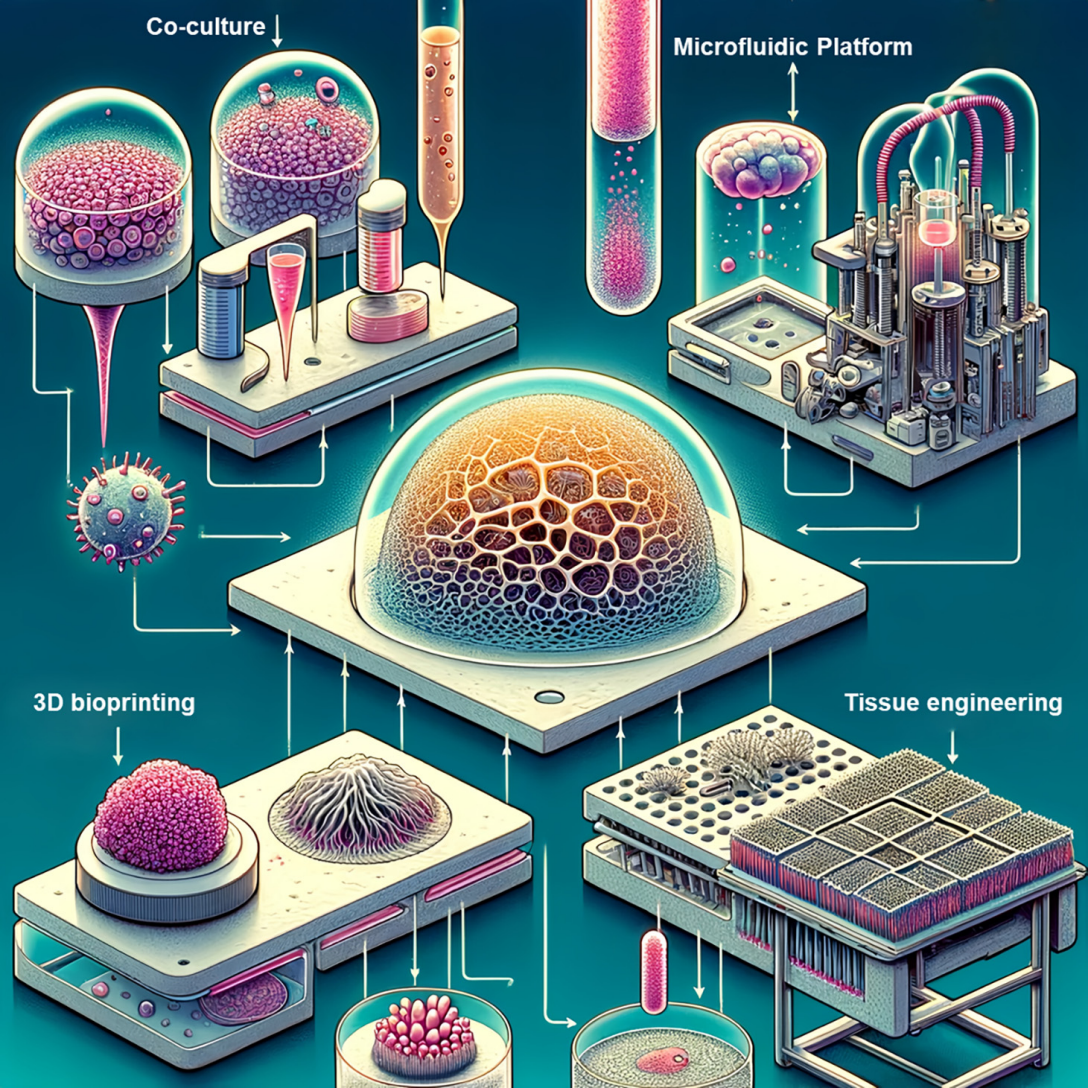
Illustration of the technology for combining organoids mimicking the heart and kidney.

The interaction between the heart and kidneys surpasses mere functional interdependence; it is synchronized through an array of biochemical signals and physiological conditions. Replicating this complex structure and function within a laboratory setting requires cutting-edge technologies and innovative methodologies (Fig. 2). Researchers are pushing the boundaries of what is possible in organoid communication as they transition beyond traditional practices into advanced co-culture methodologies. By employing these sophisticated approaches, a more physiologically relevant exchange between organoids can be established, significantly enhancing the relevance of *in vitro* models for studying complex inter-organ relationships and disease mechanisms.

The interplay between the heart and kidneys goes beyond mere functional interdependence; it is synchronized according to numerous biochemical signals and physiological conditions. Replicating this complex structure and function in a lab setting demands cutting-edge techniques and innovative methodologies.

### A. Advanced co-culture methodologies: Facilitating organoid communication

Traditional co-culture systems, capable of placing two types of organoids in close proximity, fall short in reproducing the true complexity of inter-organ communication. They lack the sophistication needed to simulate the intricate feedback mechanisms that regulate the organ function. Hence, new co-culture techniques must be designed to bridge this gap, allowing not just for heart and kidney organoids to coexist side by side but also to actively participate in a kind of mutual signaling that occurs in living organisms.

In such an enhanced co-culture environment, the transmission of signaling molecules crucial for organ function regulation, such as hormones, cytokines, and exosomes, must be meticulously engineered. For instance, the release of cardiac hormones like atrial natriuretic peptide (ANP) from heart organoids should be detectable by kidney organoids, which in turn should initiate a series of renal responses that reflect the physiological regulation of blood pressure and fluid balance.^51^ Moreover, the development of shared culture media is a critical aspect of this replication. This medium should be composed of the precise concentrations of nutrients, ions, and oxygen needed by both organ types to simulate the homeostatic environment of blood flow. This shared medium becomes a conduit for the transfer of metabolic byproducts that influence the function of the heart and kidneys in a living system. For example, the accumulation of urea and other waste products in the medium could affect the behavior of heart organoids, much like in the body, potentially inducing vital stress responses important for research.

Bestowing synchronized function between heart and kidney organoids *in vitro* requires a delicate effort. As researchers must regulate the flow of life-sustaining substances and signaling molecules, it demands not only biological expertise but also an engineering acumen. This complex interaction reflects the physiological harmony necessary for health, and its disruption can signify disease. By mastering the replication of organ interactions in the laboratory, scientists will open new horizons in understanding how our organs cooperate and function together and what happens when this harmony is lost.

### B. Microfluidic platforms: Emulating physiological blood flow

The technological advent of microfluidic platforms has revolutionized the way researchers emulate organ physiology in a controlled laboratory setting.^52–54^ These platforms are sophisticated devices designed to replicate the intricate blood flow dynamics that naturally occur between organs within the human body.^55^ The microengineering of these systems allows them to mimic the circulatory connections between organs, such as the heart and kidneys, providing an environment that is far more representative of *in vivo* conditions compared to traditional static cultures.^56^

To illustrate this with specificity, let us consider a microfluidic device engineered to model the heart and kidney interaction. This device, often called a “heart–kidney-on-a-chip,” would consist of two main microchambers or channels, each one designated to house organoid cultures of the heart and kidney. The channels are connected by a series of microscale pumps and valves that control the flow of culture medium between them, simulating the pulsatile blood flow generated by the heart. The heart organoids would be exposed to mechanical forces induced by these microfluidic pumps, which can be programmed to mimic the rhythmic beating of the heart. This mechanical stimulation is crucial, as it influences the maturation and functionality of cardiac tissues. On the downstream side, the kidney organoids receive the culture medium that has been conditioned by the heart organoids, containing signaling molecules, metabolic by-products, and oxygen levels that reflect those found in blood after it has passed through the heart.

Researchers can adjust the flow rate within the microfluidic device to model different physiological and pathological conditions. For example, they might simulate the high blood pressure of hypertension by increasing the flow rate, observing how the heightened mechanical stress affects heart organoid function and how the kidney organoids respond to the consequent changes in the signaling milieu. Conversely, conditions of low blood flow, akin to heart failure, can also be modeled to study their effects on kidney health and function. The microfluidic platforms can be integrated with sensors that continuously monitor changes in the microenvironment, such as pH, oxygen levels, and the concentration of specific biomolecules. Such real-time data allow for immediate assessment of the organoids' responses to various stimuli or interventions. For instance, introducing a cardiotoxic drug into the system could show its immediate impact on heart organoid contraction rates, followed by any resultant toxic effects on kidney organoid function, closely imitating what might happen in a human body.

In conclusion, microfluidic platforms will serve as pivotal tools in replicating the hemodynamics of human organs. Through these, it will be possible to study the individual and collective responses of heart and kidney organoids to physiological stimuli as well as to test pharmacological agents and investigate disease processes in highly controlled and physiologically relevant systems. With this technological leap, scientists will be better equipped to understand the complexities of human organ interactions and will unlock tremendous potential for advancing personalized medicine and drug development.

### C. Three-dimensional (3D) bioprinting technology: Achieving anatomical and functional fidelity

The field of tissue engineering has been significantly propelled forward by the advent of 3D bioprinting technology, a method that allows the construction of complex, multicellular structures with precision and specificity.^57–62^ 3D bioprinting leverages computer-aided design to meticulously deposit cells, along with supportive biomaterials, layer-by-layer, to form tissue constructs that closely mimic the natural architecture of human organs. In the context of creating heart–kidney-connected organoids, 3D bioprinting stands out for its ability to replicate the anatomical intricacies and functional aspects of these organs. To better understand the potential and operation of this technology, let us delve into the specifics of how 3D bioprinting could be used to create a model that includes both heart and kidney components.

First, the process begins with a detailed map or blueprint of the organ structures to be printed. This involves imaging data, such as MRI or CT scans, which can be used to design the 3D model. For the heart, this would include the complex arrangement of cardiac muscle fibers, blood vessels, and the intricate valve structures. For the kidney, it would involve the detailed nephron structures responsible for filtration and waste removal. With the blueprint in hand, scientists use a bioprinter, which is akin to a traditional 3D printer but is designed to handle biological materials. The bioprinter precisely deposits various types of cells and hydrogels—the latter acting as a scaffold to support and nurture the cells. These bio-inks are selected based on their biocompatibility and properties that support cell growth and differentiation.^63,64^ For example, to print a heart tissue, cardiomyocytes (heart muscle cells) would be mixed into a bio-ink formulation that promotes their survival and ability to contract. Similarly, for kidney tissues, renal cells would be combined with a matrix that facilitates filtration and reabsorption functions. The printer head moves in three dimensions, depositing the cell-laden hydrogel according to the design parameters, thus building up the tissue layer-by-layer.

One of the most critical aspects of 3D bioprinting for organoids is the ability to create vascular-like structures within the tissues.^65,66^ This is essential for the delivery of nutrients and oxygen to all the cells within the organoid, particularly as the structure increases in size. In the heart, this would involve printing microvascular networks that mimic coronary circulation, while in the kidney, a similar approach would need to replicate the intricate blood vessels feeding the glomeruli and nephrons. The resulting bioprinted tissues are then cultured in bioreactors where they mature and the cells begin to perform their specific functions.^67^ With time, the hydrogel scaffolds degrade, and cells start to secrete their own extracellular matrix, resulting in a self-supporting tissue.^68^ The heart tissue begins to beat synchronously, while the kidney tissue engages in filtration and secretion activities.^69,70^

By enabling the precise placement of different cell types and the construction of intricate vascular networks, 3D bioprinting provides organoids with both the form and function required to emulate the natural organ systems. This is particularly powerful for modeling diseases, testing drugs, and potentially in the future, creating tissues for transplantation that are tailor-made for individual patients. As the technology evolves, it promises to bring us closer to the creation of fully functional organ systems for a variety of biomedical applications, enhancing our understanding of human biology and advancing the possibilities of regenerative medicine.

### D. Monitoring and feedback mechanisms: Capturing real-time organoid dynamics

In the cutting-edge realm of heart–kidney organoid research, the role of monitoring and feedback mechanisms is indispensable, allowing for a nuanced appreciation of the organoids' living conditions and interactions. These systems employ an array of biosensors and advanced imaging technologies to continuously track the growth, function, and inter-organ communication, providing a stream of real-time data. The heart's rhythmic pulsations and the kidney's filtration dynamics are among the critical functions scrutinized. Microfluidic platforms play a pivotal role, not only housing the organoids but also simulating vascular responses through integrated feedback loops that can, for example, alter nutrient delivery in response to organoid activity, akin to how blood flow would naturally adjust in the body.

Complementing these hardware innovations, sophisticated software algorithms digest and interpret the data, offering predictive insights and facilitating the adjustment of conditions to mirror physiological states. Machine learning takes this a step further by refining culture conditions and predicting outcomes based on learned patterns from organoid behavior. By integrating real-time functional data from both heart and kidney organoids, researchers can derive a holistic view of organ interplay, which is instrumental in understanding how these organs would jointly function within a living system.

Despite the technological strides, the challenge remains to miniaturize sensors to fit organ-on-chip systems and to create nontoxic, biocompatible materials that can integrate into the organoids without affecting their growth or function. Looking forward, these monitoring systems will likely become more sensitive, capturing even the most subtle changes within organoids, which will be crucial for detecting early disease states and understanding the mechanisms of organ failure. The advancements in this domain not only hold promise for translational medicine but also for revolutionizing the therapeutic landscape, moving us closer to the era of personalized medicine and targeted treatments. Table II shows the innovative techniques of heart–kidney-connected organoids.

**TABLE II.** Innovative techniques and potential benefits of combining organoids that mimic the heart and kidneys.

Technique/methodology	Description	Potential benefits
Co-culture techniques	Growing heart and kidney organoids in a shared medium	Exposes organoids to the same biochemical conditions, facilitating more realistic interaction
Microfluidic platforms	Chip technologies that simulate blood flow between organoids	Emulates the actual connection between the heart and kidneys, allowing for physiological interactions
3D bioprinting	Precisely positioning heart and kidney cells in a spatially relevant manner	Enables physical and biochemical interactions that closely mirror the *in vivo* environment
Tissue engineering	Designing and creating biological tissues through a combination of cells, scaffolding materials, and biologically active molecules	Provides a platform for creating realistic, functional tissues and offers potential for therapeutic applications, like transplantable tissues
Drug testing	Exposing interconnected organoids to potential therapeutic agents	Accurate evaluation of drug effects on both organs simultaneously and leads to safer drug candidates
Disease modeling	Introducing disease-specific factors to study the pathophysiology of cardiorenal disorders and other comorbidities	Enables a deeper understanding of disease mechanisms, especially in cases where multiple organs are affected
Reduction in animal testing	Using organoids as primary models for experimentation	Enhances ethical considerations in research by potentially reducing the need for animal models

## V. BROAD APPLICATIONS AND THE FUTURE OF DRUG DISCOVERY

The evolution of biomedical research often hinges on tools that allow for nuanced exploration, and the heart–kidney-connected organoids promise to be one such transformative instrument. Their utility, while evident in studying cardiorenal syndromes, offers vast potential in broader applications that could reshape multiple facets of biomedical sciences.

### A. Advancing multi-organ disease research: The integral role of heart–kidney-connected organoids

Heart–kidney-connected organoids have emerged as a pivotal tool for advancing our understanding of complex multi-organ diseases. These diseases, such as cardiorenal syndrome, are characterized by the interdependent dysfunction of the heart and kidneys.^71^ Studying these conditions has been challenging due to the intricate nature of the feedback mechanisms and signaling pathways that connect these two vital organs.^72^ By offering a combined model of both heart and kidney tissues derived from human pluripotent stem cells, heart–kidney-connected organoids provide a unique opportunity to study the pathophysiological interplay in a controlled environment. This integrative model allows for the examination of how diseases originate and progress, taking into account the communication between the heart and kidneys that is often critical in the manifestation and progression of diseases.

The insights gained from these organoids are manifold. Researchers can observe how pathological changes in one organ, such as the heart's response to stress or injury, affect the function of the other organ, like the kidneys' filtration rate. For instance, they can investigate the impact of heart failure on kidney tissue, examining changes in renal blood flow, inflammatory responses, and fibrotic processes that may contribute to kidney disease. Additionally, these organoids enable the study of systemic responses to various stimuli, such as drug treatments or genetic modifications. Researchers can assess how modifying the expression of a gene implicated in heart disease affects kidney function or vice versa. By doing so, they can uncover novel therapeutic targets that may prevent or treat multi-organ disorders. Furthermore, heart–kidney-connected organoids facilitate the exploration of disease progression over time. Chronic conditions, which develop over an extended period, can be difficult to replicate and study in traditional models. However, organoids allow for long-term cultivation and observation, enabling researchers to monitor the evolution of disease and the long-term effects of potential therapeutic interventions.

### B. Enhancing drug development: The dual-organ assessment with heart–kidney-connected organoids

The introduction of heart–kidney-connected organoids into the drug development pipeline has the potential to revolutionize how pharmaceuticals are tested for safety and efficacy. Traditionally, drugs are assessed in isolation for each organ system, which can overlook potential cross-organ interactions that might lead to adverse side effects. Heart–kidney-connected organoids allow for a more holistic approach by providing a platform that can mimic the drug's effects on both the cardiovascular and renal systems concurrently. This dual-organ model facilitates a comprehensive analysis of a drug's impact. For instance, a new cardiac medication can be tested not only for its therapeutic effects on heart tissue but also for any nephrotoxic effects it may have. This is particularly important because many drugs that are beneficial for the heart can have unintended consequences on kidney function, and vice versa. The interconnected nature of the heart and kidneys in these organoids means that scientists can observe the downstream effects of a drug on both organ systems in real-time. Moreover, these organoids can help identify potential drug-induced organ crosstalk. Certain drugs may induce signaling pathways or release biomolecules that affect other organs. For example, a drug that leads to the release of inflammatory cytokines from cardiac cells could potentially induce stress responses in renal cells, which may only be apparent when both organ systems are present and interconnected.^73^

The ability to detect these interactions early in the drug development process is crucial. It can lead to the refinement of drug candidates before they reach clinical trials, thereby saving time and resources. The early identification of toxicities can also prevent potential harm to patients during later stages of drug testing and after the market release.

In addition to safety, the efficacy of drugs can also be more accurately gauged using heart–kidney-connected organoids. By assessing the direct effects of therapeutic compounds on organoid function, researchers can quantify improvements in organ health or amelioration of disease-related damage. This could be particularly beneficial in the development of drugs for conditions like hypertension or diabetes, where both heart and kidney functions are often compromised.

### C. Pioneering ethical alternatives: The shift to human-centric models with heart–kidney-connected organoids

The integration of heart–kidney-connected organoids into research protocols represents a significant ethical advancement in the biomedical field. These organoids offer an alternative to traditional animal testing, which has been a subject of ethical debate for many years. The use of human cell-derived organoids reduces the need for animal models, aligning scientific practices with increasing public and regulatory demand for more humane research methods.

One of the most compelling ethical arguments for using organoids is the principle of the 3Rs (replacement, reduction, and refinement) in animal research, which advocates for the replacement of animals with alternative methods, the reduction in the number of animals used, and the refinement of techniques to minimize suffering.^74^ Heart–kidney-connected organoids fulfill these criteria by potentially replacing the need for animals in certain types of research, especially those that require complex inter-organ interactions. Moreover, the shift toward human organoids can lead to a reduction in the number of animals required for experiments. Because organoids can be cultivated in controlled environments, they can be used to conduct high-throughput screenings of drugs or to study disease mechanisms with a level of precision that is often not achievable in live animals. This not only reduces the number of animals needed for conclusive studies but also improves the quality of data obtained, as the results are more directly applicable to human biology. Refinement of research techniques is also achieved with organoids. They allow for less invasive and more ethically acceptable means of studying human physiology and disease progression without subjecting animals to distressing conditions. This refinement is an ethical imperative that resonates with the broader public's concern for animal welfare.

Another significant advantage of heart–kidney-connected organoids is their potential to provide more accurate predictions of human responses to drugs and diseases. Traditional animal models, while useful, often fail to fully replicate human pathophysiology due to species differences.^75^ Organoids, however, can be generated from human cells, including those from specific patient populations, to model diseases with greater fidelity. This not only improves the translatability of research findings from the bench to the bedside but also reduces the instances of failed clinical trials due to unforeseen drug reactions that were not apparent in animal models. Furthermore, the ethical use of human-derived tissues in organoid science is underpinned by advances in stem cell technology and biobanking. Ethical sourcing of human cells, with appropriate donor consent and privacy protection, ensures that these research tools are developed responsibly. This is coupled with advancements in induced pluripotent stem cell (iPSC) technology, which allows for the generation of organoids without the need for embryonic stem cells, navigating away from another ethical concern in biomedical research.

## VI. CONCLUSION

In conclusion, this Perspective highlights the imperative need for heart–kidney-connected organoids as an invaluable tool in elucidating the dynamic and intimate connections between these organs. By bridging the knowledge gap in cardiorenal crosstalk, these integrated systems have the potential to transform biomedical research and pave the way for targeted interventions in multi-organ diseases. Our collective pursuit of this cutting-edge approach promises to unlock unprecedented insights into the complexities of human health and disease, ultimately benefiting patients worldwide.

## Data Availability

The data that support the findings of this study are available within the article.
